# Frequencies of chromosomal inversions in *Drosophila melanogaster* in Fukushima after the nuclear power plant accident

**DOI:** 10.1371/journal.pone.0192096

**Published:** 2018-02-08

**Authors:** Masanobu Itoh, Ryutaro Kajihara, Yasuko Kato, Toshiyuki Takano-Shimizu, Yutaka Inoue

**Affiliations:** 1 Department of Applied Biology, Kyoto Institute of Technology, Kyoto, Japan; 2 Advanced Insect Research Promotion Center, Kyoto Institute of Technology, Kyoto, Japan; 3 Institute of Promotion of University Strategy, Global Excellence, Kyoto Institute of Technology, Kyoto, Japan; 4 Center for Education in Liberal Arts and Sciences, Osaka University, Toyonaka, Japan; University of South Carolina, UNITED STATES

## Abstract

In order to investigate genetic impact of a large amount of radionuclides released by the Fukushima Dai-ichi Nuclear Power Plant accident in 2011, we surveyed 2,304 haploid genomes of *Drosophila melanogaster* collected in three localities in Fukushima in 2012 and 2013 for chromosomal inversions. No unique inversion was found in 298 genomes in 2012 and only two in 2,006 genomes in 2013. The observed frequencies were even lower than the long-term average frequency of unique inversions in Japan. The common cosmopolitan inversions were also examined in Fukushima, Kyoto, and Iriomote (Okinawa) in 2012. Among three samples in Fukushima, the flies in Iizaka, where environmental radiation level was the highest, showed the lowest frequency of *In(2L)t*, but the highest frequency of *In(3R)P*, contrary to the expectation of decreasing of their frequencies in higher polluted areas. These results suggest that, at this level of genetic analysis, Fukushima populations of *D*. *melanogaster* would not have been negatively impacted following the release of radionuclides. Transposable *P*-element mobility was not likely to induce DNA damage solely or synergistically with radioactivity, because their transposition activity was totally repressed in the Fukushima strains. However, it should be noted that, because of limitations in access to the exclusion zone, we could only sample the populations in areas of relatively low radioactive contamination (0.39–0.63 μSv/h). Therefore, the present study is likely to be underpowered to detect any effects that might be expected in heavily contaminated areas.

## Introduction

Meltdown of the three reactors of Fukushima Dai-ichi Nuclear Power Plant, caused by the Great East Japan Earthquake on March 11, 2011, resulted in releasing a large amount of radionuclides. Because of the mutagenic effects of ionizing radiation, influence of environmental radioactive contaminants on living organisms has been of great concern worldwide [1, 2]. As the case of Chernobyl (for a review, [3]), surveys of non-human biota in the general area of the disaster are important consideration in an eventual understanding of biological outcomes of the accident [4–6]. Several studies have reported increased biological changes in Fukushima such as morphological abnormalities in the pale grass butterfly *Zizeeria maha* [7] and the barn swallow *Hirundo rustica* [8], reduced hematological values in the wild Japanese monkey *Macaca fuscata* [9], and shrinking of biodiversity in insect and bird species [10–13] to date. However, except for DNA damages shown by comet assay in *Magascolecidae* earthworms [14] and an increase of dicentric chromosomes in wild mice *Apodemus argenteus* and *Mus musculus* [15], convincing evidence for genetic effects has not yet been obtained in the Fukushima accident.

Drosophila has been used as a standard tester organism for analyzing the impact of the environmental radionuclides pollution, for instance, in the Marshall Islands including Bikini [16] and Chernobyl [17, 18]. *D*. *melanogaster*, one of the most well-studied model organisms, is especially suitable for this purpose because of their distribution in almost all temperate and tropical regions of the world and favorable breeding nearby human habitats [19, 20]. Many early studies on sex-linked lethal mutations found a linear dose-response relationship in *D*. *melanogaster* [21], although effects of low-dose irradiation for many generations remain elusive [22–25]. Chromosomal inversion frequency could also be an index of the impact of radiation. DNA double strand breaks (DSBs), which are caused by ionizing radiation, can lead to production of inversions[26, 27]. It was experimentally shown that 40 Gy of X-ray irradiation induces chromosomal inversion at the rate of 0.007 or greater. Such *de novo* inversions should be identified as unique inversions [28].

The four common cosmopolitan inversions, *In(2L)t*, *In(2R)NS*, *In(3L)P*, and *In(3R)P*, are known to show the latitudinal clines in frequency, lower frequencies in higher latitude in both of Northern and Southern hemispheres [29–31]. Their frequencies may also be affected by a deleterious mutation rate in the population. Marked decreases in frequency have been previously observed in a population of Katsunuma, Japan, 0.32 to 0.11 for *In(2L)t*; 0.21 to 0.12 for *In(2R)NS*; 0.09 to 0.02 for *In(3L)P*, from the late 1960s to the middle of 1970, while the frequency of *In(3R)P* fluctuated largely, but did not change eventually in the period [32, 33]. These inversions are hypothesized to be maintained through heterosis or epistatic selection in an environment [34–36] and deleterious mutations may affect more severely the fate of such inversion chromosomes [37]. Mukai and his colleagues suggested that the decrease in the inversion frequencies in Katsunuma would be associated with a temporary higher mutation rate caused by *P*-element transposition [38]. *P*-elements are DNA transposons that invaded *D*. *melanogaster* world-wide populations in the middle of the 20th century [39–42]. In the P-M system, P strains carry many genomic copies of *P*-elements and M strains have no copy. *P*-elements transpose in the germline of F1 progeny between M females x P male and lead to P-M hybrid dysgenesis containing sterility (gonadal dysgenesis), elevated mutation rate, and male recombination (for reviews, see [19, 43]). *P*-elements are also known to induce DNA damage synergistically with radioactivity [44, 45].

The previous frequencies of chromosomal inversions in Fukushima are fortunately available for the unique inversions in Japan from 1975 to 1983 [28] and the common cosmopolitan inversions in Fukushima in 2009 [46], so that one can use them as base lines for comparisons. Here, we surveyed the frequencies of the common cosmopolitan and unique inversions in 1,152 lines of *D*. *melanogaster* collected in Fukushima in 2012 and 2013 to evaluate the genetic impact of the Fukushima accident. We also performed the GD sterility tests in the P-M system [47, 48] to measure the activity of *P-*elements as one of the possible mutagens in Fukushima.

## Materials and methods

### Fly collection

*D*. *melanogaster* flies were collected by banana traps or by net-sweeping over Drosophila-attractive sites in five localities of Japan from 2012 to 2013 (Table 1). Collection points of FIZ, MSH, and MSO are from 68, 25, and 16 km away from the Fukushima Dai-ichi Nuclear Power Plant, respectively. Because the government limited to access to the exclusion zone, Odaka in Minami-Soma (MSO) was one of the closest towns to the Power Plant where citizens were allowed to visit in July, 2013. Additionally, the fly population inside the Plant was expected too small to compare the frequencies of the inversions, because the Plant is located very at the shore, where *D*. *melanogaster* does not favorite to dwell. After collection, females were kept individually in vials to establish isofemale lines. Flies were maintained with standard corn-sugar-yeast medium (3% cornmeal, 4% dried yeast, 10% glucose, 0.7% agar, 0.5% propionic acid, and 0.5% butyl-p-hydroxybenzoate) at 25°C.

**Table 1:** Sample collection.

			Radio activity (μSv/h)			
Symbol	Location	Lat. °N, Long. °E	Ave.	S.D.	n*	Collection time
FIZ	Iizaka, Fukushima	37.80, 140.40	0.636	0.17	11	June, 2012 and July, 2013
MSH	Haramachi, Minami-Soma	37.64, 140.96	0.422	0.241	11	June, 2012 and July, 2013
MSO	Odaka, Minami-Soma	37.56, 140.99	0.391	0.081	8	Oct., 2012
KY	Sakyo, Kyoto	35.04, 135.78	0.06**			Nov., 2012
IR	Iriomote, Okinawa	24.40, 123.81	0.01**			Dec., 2012

* Number of surveying points

** Data from the radioactivity map by Fukushima Prefecture (http://fukushima-radioactivity.jp/pc/)

### Assessing ambient radiation levels

We measured radiation levels by using a beta (gamma) survey meter (model TGS-136, ALOKA) at each collecting point of the flies in Fukushima in November 2013. The detector was aimed at the ground at 1 m high. The doses did not largely contradict the public report (http://www.safecast.jp/maps/ or http://www.Fukushima-radioactivity.jp). Radiation levels of Kyoto and Iriomote island were quoted from above databases.

### Karyotyping

We determined karyotype of each isofemale line by using the chromosomal squash technique and phase-contrast microscopy [28]. Briefly, one third-instar larva from each isofemale line was dissected and the salivary gland chromosomes were stained with lactic-acetic orcein. The banding patterns were compared with the standard map of the salivary gland chromosomes of *D*. *melanogaster* [49] and the karyotype of the line was determined according to the previous reports [46, 50]. The karyotyping was carried out from June 2012 to February 2013 for the 2012 samples and from July to December 2013 for the 2013 samples.

The *In(2L)t* and *In(2R)NS* genotypes were also determined by PCR typing with the primers amplifying both standard and inversion chromosomes [51, 52]. We randomly picked up 16 or more isofemale lines for each locality-year sample and extracted genomic DNAs individually from four adult flies per each isofemale line by using the GeneElute Mammalian Genomic DNA miniprep kit (Sigma-Aldrich). The PCR reaction condition for *In(2L)t* was as follows: 31 cycles of denaturing at 95°C for 30 sec, annealing at 60°C for 30 sec, and polymerizing at 72°C for 1 min. As for typing of *In(2R)NS*, we carried out a pair of PCR, one for amplification of *In(2R)NS* (31 cycles of denaturing at 98°C for 10 sec, annealing at 60°C for 30 sec and polymerizing at 72°C for 90 sec) and the other for that of the standard chromosomes (31 cycles of denaturing at 95°C for 10 sec, annealing at 60°C for 30 sec and polymerizing at 72°C for 90 sec). ExTaq (Takara) was used as the enzyme for amplification.

### Gonadal dysgenesis (GD) tests

According to the standard method of GD test in the P-M system [47, 48], we performed a set of diagnostic crosses, cross A (M females x tested males) and cross A* (tested females x P males), at 29°C, using Canton S as a reference M and Harwich as a P strain. We individually dissected more than 40 F1 females and calculated the GD score (GD%) for each line as the frequency of undeveloped ovaries. The GD% score in cross A indicates the inducing ability and that in cross A* indicates the susceptibility of *P*-element transposition. The strain Harwich was obtained from Kyoto Drosophila Stock Center.

## Results

### Radiation levels in collection localities

Among five localities in Japan, the radiation levels in Fukushima (FIZ, MSH, and MSO), 0.39–0.64 μSv/h, were considerably higher than in Kyoto and Okinawa (Table 1). The highest level was detected in FIZ in Fukushima.

We measured the radiation levels at several other points; the average was 0.45 μSv/h (0.06–1.05, n = 6) in Fukushima City, 1.3 μSv/h (0.33–1.88, n = 7) road side between MSH and MSO, and 2.2 μSv/h (1.75–2.48, n = 3) in Iidate Town. Unfortunately, we could not obtain enough number of flies in these sites.

### Inversion chromosomes in the 2012 samples

We assessed the inversion frequencies in 492 genomes in the above five localities in 2012 (Table 2). One unique inversion, *In(2R)46B*:*55F*, was found in Kyoto (122 genomes), but neither in Fukushima (298 genomes) nor in Iriomote (72 genomes). Three of the four common cosmopolitan inversions, *In(2L)t*, *In(2R)NS*, and *In(3R)P*, were commonly observed in all localities, while *In(3L)P* was only observed in Kyoto and Iriomote. Two rare cosmopolitan, *In(3R)C* and *In(3R)Mo*, and one endemic, *In(2R)O*, inversions were also found in some localities. Additionally, we estimated the *In(2L)t* and *In(2R)NS* frequencies by PCR typing with more than one hundred genomic DNAs for each sample and the obtained frequencies (S1 Table) were not in contradiction with those from the microscopic observation.

**Table 2:** Frequencies of inversion chromosomes in natural populations.

			2L	2R			3L	3R			
Sample	Year	n	*t*	*NS*	*O*	unique***	*P*	*P*	*C*	*Mo*	unique***
Fukushima09*	2009	92	0.083	0.022	0	0	0	0.065	0	0	0
FIZ12	2012	112	0.036	0.009	0	0	0	0.134	0	0	0
MSH12	2012	74	0.122	0.041	0	0	0	0.068	0	0	0
MSO12	2012	112	0.107	0.045	0.009	0	0	0.089	0	0	0
Fukushima12**	2012	298	0.084	0.030	0.003	0	0	0.101	0	0	0
KY12	2012	122	0.165	0.157	0.05	0.016	0.008	0.107	0.074	0.025	0
IR12	2012	72	0.444	0.236	0.083	0	0.347	0.444	0	0	0
FIZ13	2013	1006	0.0815	0.0189	0	0	0.003	0.1173	0	0.001	0
MSH13	2013	1000	0.103	0.022	0.006	0.001	0.003	0.063	0	0	0.002

n: number of genomes examined.

* Data from the previous paper [26].

** Combined by the three samples of in Fukushima in 2012, FIZ12, MSH12, and MSO12.

*** 46B:55F in KY12, 42A:59D in MSH13, and 87C:89F in MSH13.

**Fig 1:**
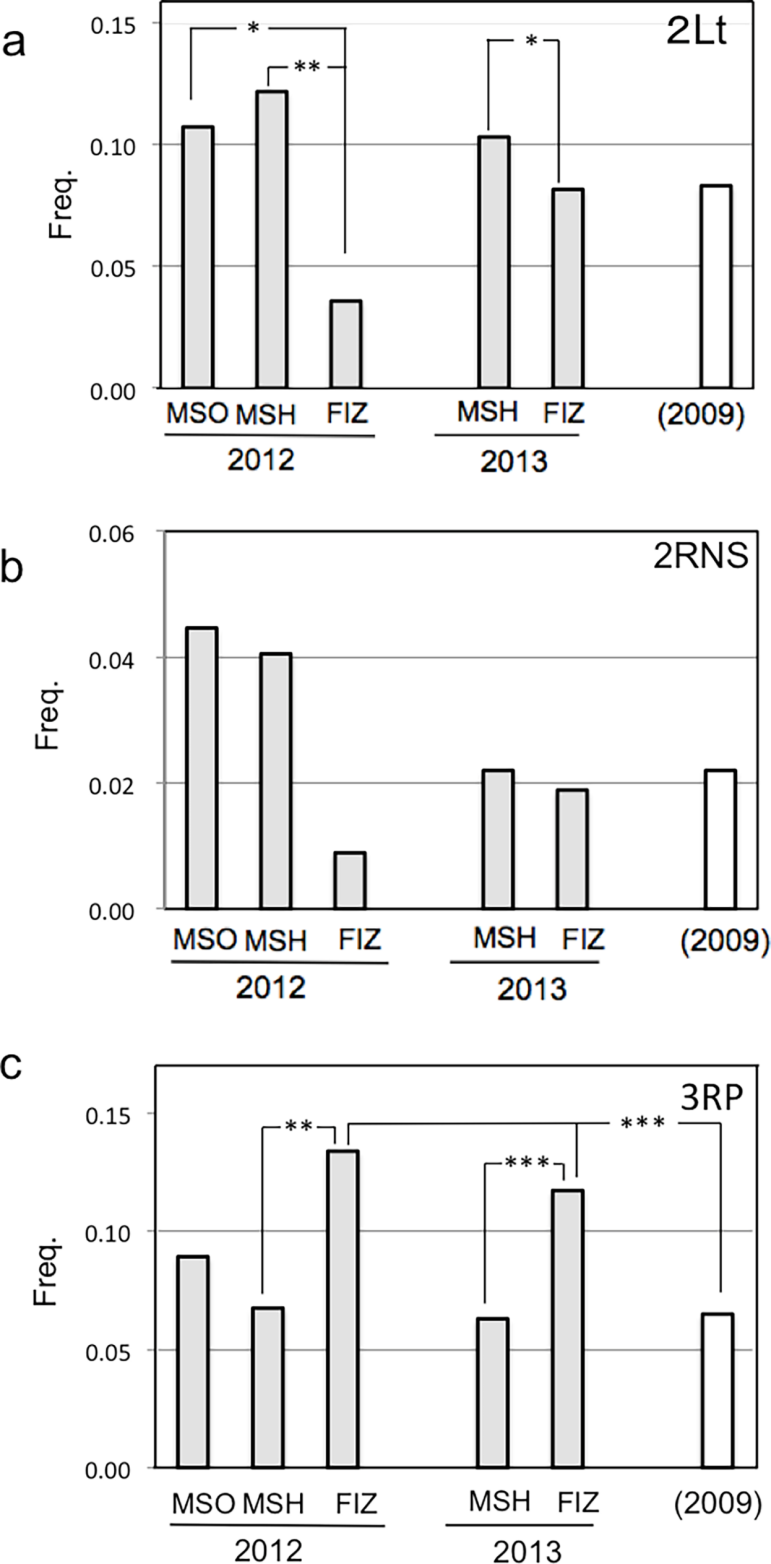
Frequencies of common cosmopolitan inversions in Fukushima. (a) *In(2L)t*, (b) *In(2R)NS* and (c) *In(3R)P*. Data of 2009 are cited from the previous report [46]. Significance of difference in Chi square test are depicted by * (*p* < 0.05), ** (*p* < 0.01), or *** (*p* < 0.001).

Among the three localities in Fukushima, the frequency of *In(2L)t* was significantly lower in FIZ12 than in MSH12 (*X* ^2^ = 7.78, *p* < 0.01) and MSO12 (*X* ^2^ = 5.96, *p* < 0.05) (Fig 1A). The same tendency was seen in *In(2R)NS*, although not statistically significant (Fig 1B). In contrast, the frequency of *In(3R)P* was significantly higher in FIZ12 than in MSH12 (*X* ^2^ = 7.68, *p* < 0.01) and that in 2009 (*X* ^2^ = 8.76, *p* < 0.01) (Fig 1C). When the data of the three localities in Fukushima, FIZ12, MSH12, and MSO12, are combined as Fukushima12, the inversion frequency was the highest in Iriomote and the lowest in Fukushima for all four common cosmopolitan inversions, suggesting the persistence of their original latitudinal cline [29] in this range (Table 2).

### Inversion chromosomes in the 2013 samples

In order to know the frequency of *de novo* inversions in the Fukushima area, we further examined a total of 1,003 isofemale lines of MSH13 and FIZ13. Only two new types of inversions, *In(2R)42A*:*59D* and *In(3R)87C*:*89F*, were found in MSH13, but not in FIZ13 (Table 2). In sum, the ratio of autosomal unique inversion per haploid genome was 0.0015 (3/2006) in Fukushima in 2013.

All four common cosmopolitan inversions were detected in both samples (Table 2). The frequency of *In(2L)t* was slightly lower in FIZ13 than in MSH13 (*X* ^2^ = 5.03, *p* < 0.05), while the frequency of *In(3R)P* was significantly higher in FIZ13 than in MSH13 (*X* ^2^ = 50.4, *p* < 0.001) and in Fukushima09 sample (*X* ^2^ = 65.3, *p* < 0.001). There was no significant difference in the frequency of *In(2R)NS* between FIZ13 and MSH13, or between the current and previous samples. The frequency of *In(3L)P* was prominently lower than others.

### Transposition activity of *P*-elements in Fukushima

To know the current transposition activity of *P-*elements as one of the mutagens in Fukushima, we carried out GD test for MSH12, MSO12, FIZ12, and KY12. The average of GD% in the crosses A and A* were both less than 10% in the three samples in Fukushima (Table 3). Almost all lines were Q-type strains, which have low potential of transposition and low susceptibility of *P-*elements [43], in Fukushima, with only one exception, FIZ12-11, showing a higher susceptibility (S2 Table). Each line of KY12 was also shown to have low inducibility and low susceptibility (Table 3, and S3 Table).

**Table 3:** P-M gonadal dysgenesis in the Fukushima population.

		cross A GD%		cross A* GD%	
Sample	n	Ave.	St Dev.	Ave.	St Dev.
MSH12	12	1.71	2.73	0.54	0.76
FIZ12	13	0.15	0.55	7.62	22.29
MSO12	7	1.48	2.06	3.24	4.95
KY12	10	5.14	10.52	2.19	3.71

The GD% in cross A indicates the inducing ability and that in cross A* indicates the susceptibility of *P-*elements transposition.

n: number of isofemale line examined.

Results of the each line are shown in S2 and S3 Tables, in detail.

## Discussion

An average frequency of unique inversions was previously shown as 0.0080 in Japan by the survey of 10,046 lines collected in 31 localities from 1975 to 1983 [28]. The current frequency in Fukushima in 2013, 0.0015, was smaller than this (X ^2^ = 10.7, *p* = 0.011). Even if limited to MSH13 only, the value of 0.003 was less than half of the long-term average. The frequency of *de novo* inversion was expected to increase, if the inversion induction rate would increase and some of such inversions would be maintained from 2011 to 2013. However, this was not the case in Fukushima. Our present result suggests that there has been no recent increase in the frequency of unique inversions in the Fukushima.

As for the common cosmopolitan inversions, there were some differences in their frequencies between samples (Table 2). FIZ showed the lowest frequencies of *In(2L)t* and *In(2R)NS* in both 2012 and 2013, although difference of *In(2R)NS* was not statistically significant (Fig 1A and 1B). On the other hand, the frequencies of *In(3R)P* in FIZ12 and FIZ13 were both significantly higher than that before the accident (Fig 1C). FIZ showed higher environmental radioactivity in average than MSO or MSH. The tendencies of the inversion frequencies observed in FIZ interestingly accord with those observed in Katsunuma during 1960s and 1970s, namely decreasing of *In(2L)t* and *In(2R)NS* and a large fluctuation of *In(3R)P*. Therefore, the difference in the frequencies of *In(2L)t* and *In(2R)NS* between localities or collection times could be associated with the radiation activity. However, no significant difference was detected between the 2009 sample and any of the present samples in the frequencies of *In(2L)t* or *In(2R)NS* (Fig 1A). In addition, all observed changes including that of *In(3R)P* do not conflict to persistence of their original latitudinal cline in the regions, Fukushima, Kyoto, and Okinawa. Stochastic fluctuation would be one of the alternative explanations of the differences observed. Moreover, the GD test showed that transposition activity of *P*-elements was totally repressed, if any, in Fukushima. Therefore, it is unlikely that *P*-element mobility induced DNA damage solely [38] or synergistically with radioactivity [44, 45] in Fukushima.

Zainullin and the colleagues examined *D*. *melanogaster* populations near the Chernobyl nucleic power plant from 1986 to 1989 [17]. They reported that lethal X chromosome were found at prominently higher frequencies, 0.0104 in Red Forest (2 km away from the power plant, >2400 μSv/h of the ambient radiation level) in 1987 and 0.0036–0.0053 in a collection point named Chernobyl (14 km apart, 60 μSv/h) in 1986, implying a strong genetic impact of environmental irradiation on the *Drosophila* populations. Although the current ambient levels remain in the 10–1000 μSv/h range in Red Forest, even more than 30 years later[53, 54], the levels in 1989 were reported as 0.2–2 μSv/h in Lubyanka (15 km apart), Ylntsi (15 km apart), and the Chernobyl, where the frequency of lethal chromosomes decreased to a control level, less than 0.0019[17]. The ambient levels of these three points are interestingly overlapped to those observed in Fukushima in this study, 0.39–0.64 μSv/h in 2013, despite of a quite large differences in the amount and nuclear species released to environment between the cases of Fukushima and Chernobyl[10, 55].

Together all, we could not obtain clear empirical evidence for a major genetic impact of radioactive pollution on *D*. *melanogaster* from the frequencies of the inversion chromosomes in Fukushima area. However, our results should be interpreted with caution because the sampling sites were limited and they were outside of the regions of highest contamination in Fukushima. Indeed, the ambient radiation levels are at the lowest ends of the range of contamination observed around Chernobyl. A comprehensive understanding of genetic impacts of ionizing radiation on living organisms and endemic populations in Fukushima will require further survey of the Drosophila populations with a larger and more representative population sample, especially from extremely contaminated zone and the surroundings.

## Supporting information

S1 Table Number of individuals and frequencies of inversion chromosomes by PCR. (XLSX)

S2 Table Results of GD test of natural population in Fukushima in 2012. (XLSX)

S3 Table Results of GD test of natural population in Kyoto in 2012. (XLSX)
